# How to Remove Amalgam Fillings

**Published:** 1861-02

**Authors:** 


HOW TO REMOVE AMALGAM FILLINGS.—A short time ago I met Dr. E. Meader of Bermuda, formerly of New York. During our conversation we naturally came to speak of our profession, in the course of which he said to me that during his residence in Bermuda he was often called upon to remove Amalgam Fillings, and to replace them with gold ones. This operation is a tedious one, as it is well known to every dentist; therefore, he came to the idea that perhaps by applying some mercury to the surface of the filling, previously well cleaned, it would become reabsorbed, and would in a few minutes time soften the old amalgam. Having made a trial, he found it to answer his most sanguine expectations. He used it in the following way. He first made a little tube with wax to act as a conducting channel for the mercury, this being filled up with a little mercury, and the surface of the filling being well cleaned and scraped, he rubbed upon it a little diluted sulphuric acid, which would favor the amalgamation, then, when all is ready, he applied his tube filled with mercury to the surface of the filling. Having tried this, I have found it of inestimable value in removing these execrable remnants of a past age.—*New York Dental Journal.*

